# Partitioning drivers of spatial genetic variation for a continuously distributed population of boreal caribou: Implications for management unit delineation

**DOI:** 10.1002/ece3.4682

**Published:** 2018-12-14

**Authors:** Pauline Priadka, Micheline Manseau, Tim Trottier, Dave Hervieux, Paul Galpern, Philip D. McLoughlin, Paul J. Wilson

**Affiliations:** ^1^ Natural Resources Institute University of Manitoba Winnipeg Manitoba Canada; ^2^ Landscape Science and Technology Division Environment and Climate Change Canada Ottawa Ontario Canada; ^3^ Biology Department Trent University Peterborough Ontario Canada; ^4^ Ministry of Environment Saskatchewan Government La Ronge Saskatchewan Canada; ^5^ Department of Environment and Parks Alberta Government Grande Prairie, Alberta Canada; ^6^ Faculty of Environmental Design and Department of Biological Sciences University of Calgary Calgary Alberta Canada; ^7^ Department of Biology University of Saskatchewan Saskatoon Saskatchewan Canada

**Keywords:** clinal differentiation, genetic connectivity, landscape genetics, Moran’s eigenvector maps, population structure, *Rangifer tarandus caribou*

## Abstract

Isolation by distance (IBD) is a natural pattern not readily incorporated into theoretical models nor traditional metrics for differentiating populations, although clinal genetic differentiation can be characteristic of many wildlife species. Landscape features can also drive population structure additive to baseline IBD resulting in differentiation through isolation‐by‐resistance (IBR). We assessed the population genetic structure of boreal caribou across western Canada using nonspatial (STRUCTURE) and spatial (MEMGENE) clustering methods and investigated the relative contribution of IBD and IBR on genetic variation of 1,221 boreal caribou multilocus genotypes across western Canada. We further introduced a novel approach to compare the partitioning of individuals into management units (MU) and assessed levels of genetic connectivity under different MU scenarios. STRUCTURE delineated five genetic clusters while MEMGENE identified finer‐scale differentiation across the study area. IBD was significant and did not differ for males and females both across and among detected genetic clusters. MEMGENE landscape analysis further quantified the proportion of genetic variation contributed by IBD and IBR patterns, allowing for the relative importance of spatial drivers, including roads, water bodies, and wildfires, to be assessed and incorporated into the characterization of population structure for the delineation of MUs. Local population units, as currently delineated in the boreal caribou recovery strategy, do not capture the genetic variation and connectivity of the ecotype across the study area. Here, we provide the tools to assess fine‐scale spatial patterns of genetic variation, partition drivers of genetic variation, and evaluate the best management options for maintaining genetic connectivity. Our approach is highly relevant to vagile wildlife species that are of management and conservation concern and demonstrate varying degrees of IBD and IBR with clinal spatial genetic structure that challenges the delineation of discrete population boundaries.

## 1 INTRODUCTION

Genetic approaches are increasingly being applied to delineate boundaries around demographically divergent groups of individuals, often termed populations (Waples & Gaggiotti, 2006) or management units (Palsbøll, Berube, & Allendorf, 2007; Yannic et al., 2016; Zannèse et al., 2006). Whereas multiple indices exist to characterize genetic differentiation for population‐level delineation, correctly identifying population structure and drivers of genetic variation is often difficult. Central to the problem is that detection of gene flow, and consequently population structure, can be confounded by drivers operating at varying temporal and spatial scales (Anderson et al., 2010; Frantz, Cellina, Krier, Schley, & Burke, 2009; Landguth & Schwartz, 2014; Meirmans, 2012). Detecting structure at the population level is especially difficult for continuously distributed species that disperse short distances across heterogeneous landscapes, maintaining low levels of genetic differentiation that results in a clinal pattern of gene flow across large spatial scales.

Clinal genetic patterns are most commonly described by the natural pattern of isolation‐by‐distance (IBD). IBD was first described by Wright (1943) and indicates naturally decreasing gene flow based on the average dispersal range of individuals (Strien, Holderegger, & Heck, 2015). The most commonly reported problem in the literature is that strong levels of IBD can cause spurious clusters to be identified along continuous genetic clines (Frantz et al., 2009; Meirmans, 2012). The misinterpretation of spurious clusters can lead to incorrect characterization of population structure and population‐level delineation (Frantz et al., 2009; Palsbøll et al., 2007). Further, IBD can mask alternative patterns of population structure resulting from resistance to gene flow caused by landscape heterogeneity and/or environmental variables (Garnier, Alibert, Audiot, Prieur, & Rasplus, 2004; Strien et al., 2015) termed isolation‐by‐resistance (IBR). Disentangling the effects of IBD and IBR is an important step in confirming population‐level delineation and identifying potential causes of detected population structure (Guillot, Leblois, Coulon, & Frantz, 2009; Palsbøll et al., 2007). Understanding drivers of genetic connectivity across the landscape is especially important for the conservation and management of species at risk that are expected to experience range retractions and consequently loss in genetic connectivity across anthropogenically modified landscapes (Manel, Schwartz, Luikart, & Taberlet, 2003; Storfer, Murphy, Spear, Holderegger, & Waits, 2010; Strien et al., 2015).

**Figure 1:**
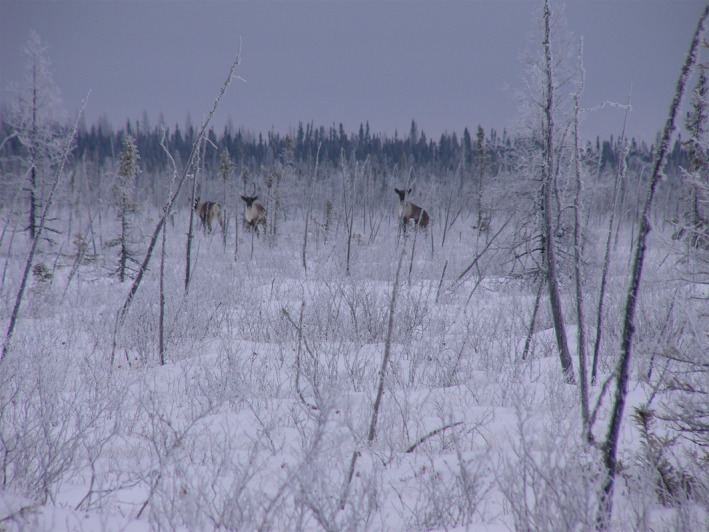
Boreal caribou (*Rangifer tarandus caribou*) is listed as threatened under the Species at Risk Act (SARA) in Canada

**Figure 2:**
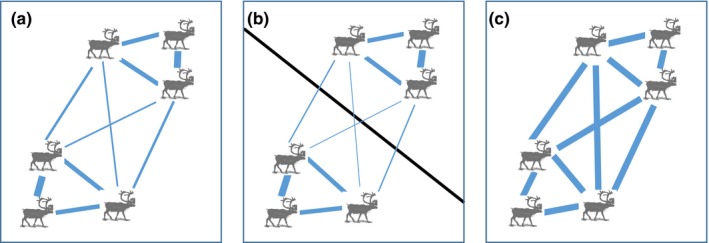
Models of processes that can shape population genetic structure, including (a) isolation by distance, (b) isolation by resistance, and (c) panmixia. Thicker blue lines indicate higher gene flow between individuals. The black line in (b) represents spatial landscape variables that can create resistance to gene flow between individuals

The boreal ecotype of woodland caribou (*Rangifer tarandus caribou*) is (Figure 1), hereafter referred as boreal caribou, is experiencing population declines across its range and is listed as threatened under the Federal Species at Risk Act (SARA, c.29, Schedule 1). A distinguishing characteristic of boreal caribou that separates them from other caribou ecotype is that they have overlapping summer and winter ranges and do not form discrete or migratory groups (Ferguson & Elkie, 2004; Festa‐Bianchet, Ray, Boutin, Côté, & Gunn, 2011). The federal recovery strategy for boreal caribou (Environment Canada, 2012) delineates “local populations” based on observation and telemetry data from collared females (Environment Canada, 2011, 2012 ) as the conservation unit to aid in monitoring and recovery efforts. Information on population genetic structure and connectivity has been sought to validate and characterize the system, to inform range and land‐use planning activities and ultimately, to preserve genetic diversity and connectivity. In western Canada, genetic connectivity of boreal caribou has been previously identified to be moderate (*F*_ST _ranging from 0.02 to 0.08) (Ball, Finnegan, Manseau, & Wilson, 2010; Klütsch, Manseau, Trim, Polfus, & Wilson, 2016) and primarily affected by roads, rivers, and large lakes (Galpern, Manseau, & Wilson, 2012; Koper & Manseau, 2009; McLoughlin, Paetkau, Duda, & Boutin, 2004). In using a more comprehensive dataset, we expect that population structure of boreal caribou will be characterized by IBD (Figure 2a) in areas presenting limited road network and discrete natural features, and by IBR (Figure 2b) where human activities and natural features (lakes/wildfires) are more prominent. We do not expect panmixia (i.e., random mating; Figure 2c) to be supported.

In this paper, we use a combination of population‐based genetic analyses including STRUCTURE (Pritchard, Stephens, & Donnelly, 2000), MIGRATE (Beerli & Felsenstein, 2001), and a spatial statistical method MEMGENE that uses Moran's eigenvector maps (Galpern, Peres‐Neto, Polfus, & Manseau, 2014) to explicitly model spatial patterns of population structure for an extensive distribution of boreal caribou in western Canada. Furthermore, to partition drivers of spatial genetic variation, we introduce an extension of MEMGENE analysis that allows comparison between the proportion of genetic variation attributed to Euclidean distance between samples (IBD) and the proportion attributed to the landscape (IBR) (Galpern & Peres‐Neto, 2014). We also apply a MEMGENE analysis that uses genetic variation partitioning to evaluate different management unit (MU) scenarios and identify population‐level boundaries that best reflect detected spatial genetic patterns to inform conservation and management planning and actions.

Our study asks three questions: (1) What model of population genetic structure best describes boreal caribou across a continuous distribution in western Canada? (2) Do IBR hypotheses associated with anthropogenic features (highways and provincial roads), waterbodies, and wildfires contribute to the observed patterns of genetic variation? and (3) What boundaries best capture the patterns of spatial genetic variation and could be used to delineate MUs for conservation purposes? We apply both aspatial and spatial methods as part of a framework for delineating management units for vagile wildlife species that present a natural clinal pattern of population genetic structure.

## 2 MATERIALS AND METHODS

### 2.1 Study area

**Figure 3:**
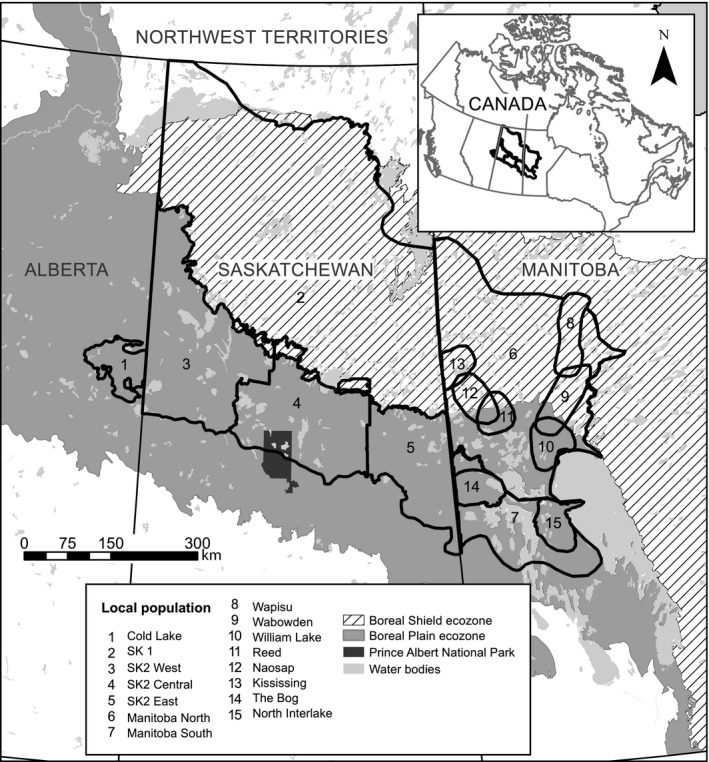
Map of boreal caribou ranges where sampling took place in the study. Ranges represent local populations that are outlined in the federal recovery strategy (Environment Canada, 2012) and updated in the provincial range plan in Saskatchewan (Saskatchewan Ministry of Environment, 2017)

Our study area includes the Cold Lake range in northeastern Alberta, and spans the Boreal Plain and Boreal Shield ecozones in Saskatchewan and western Manitoba, comprising an area of 401,645 km^2^. Local population boundaries outlined in the federal recovery strategy (Environment Canada, 2012) and updated in the provincial boreal caribou range plan in Saskatchewan (Saskatchewan Ministry of Environment, 2017) that overlap the study area are depicted in Figure 3. The landscape across the Boreal Plain is comprised of mixed deciduous and coniferous forest interspersed in lowland areas by peat bogs and muskeg (Environment Canada, 2009). The Boreal Shield, which is immediately north of the Boreal Plain in Saskatchewan and Manitoba, is dominated by pine and black spruce forests over Pre‐Cambrian Shield (McLoughlin et al., 2016). Habitat disturbance from wildfire is much more prominent in the Boreal Shield due to minimal active fire suppression and drier habitat resulting in shorter fire cycles of ~100 years (McLoughlin et al., 2016).

### 2.2 Genetic sampling

Our samples collected in the Boreal Plain ecozone in Saskatchewan relied on a large‐scale, noninvasive fecal pellet collection program carried out from 2004 to 2016 by the Government of Saskatchewan Ministry of Environment and Prince Albert National Park. Fecal pellet sampling in Manitoba was conducted by Manitoba Conservation, Parks Canada, and Manitoba Hydro from 2003 to 2006. Samples from Cold Lake range in Alberta were collected by Alberta Environment and Parks in winter 2013/14. The areas were surveyed systematically by fixed‐wing aircraft (linear transects) to identify tracks and foraging sites made by caribou in snow. A field crew subsequently visited each foraging site by helicopter to collect fecal pellets, and coordinates for each collection site were recorded. Our samples from the Boreal Shield ecozone in Saskatchewan were obtained from blood blots or vials collected from individual boreal caribou handled during radio‐collaring in the winters 2013–2014 (McLoughlin et al., 2016).

### 2.3 DNA extraction and genotyping

Extracted DNA was amplified and genotyped at nine microsatellite loci markers (BM848, BM888, MAP2C, RT5, RT6, RT7, RT9, RT24, and RT30; Bishop et al., 1994; Wilson, Strobeck, Wu, & Coffin, 1997; Cronin, MacNeil, & Patton, 2005) following procedures in Ball et al. (2007) and Klütsch et al. (2016). Data quality and genotyping error rates have been assessed and reported on in previous studies that have used some of the same data (e.g., Ball et al., 2007; Ball et al., 2010; Hettinga et al., 2012; Klütsch et al., 2016) and that found low error rates below the recommended threshold of 0.05 (Roon, Waits, & Kendall, 2005). Due to the quantity and high molecular weight DNA of the winter‐collected fecal pellets, a multitube approach was not applied. Each genotype was scored by two or three different laboratory personnel to cross‐check for errors using GENEMARKER genotyping software version 1.75 (SoftGenetics LLC). Because individual caribou may have been sampled more than once, duplicate samples were identified with a 8 out of 9 matching loci threshold using the program Allelematch version 2.03 (Galpern, Manseau, Hettinga, Smith, & Wilson, 2012) in R version 3.2.1 (R Core Team, 2015) and were removed from subsequent analyses.

### 2.4 Population genetic structure: STRUCTURE

The most likely number of genetic clusters (*K*) across the study area was determined using the individual‐based Bayesian clustering program STRUCTURE version 2.3.4 (Pritchard et al., 2000). Because admixture is expected to be high across the study area (Ball et al., 2010), we used the admixture model implemented in STRUCTURE and applied 10^6^ MCMC iterations and a burn‐in length of 50^5^ for each *K* that was set to range from 1 to 10 (Falush, Stephens, & Pritchard, 2003). Optimal *K* was chosen by evaluating likelihood of K (L(K)) and delta K (ΔK) plots (Evanno, Regnaut, & Goudet, 2005) that were produced using STRUCTURE HARVESTER web version 0.6.94 (Earl & vonHoldt, 2012). Individual assignment to a cluster (q) was determined by averaging across five iterations for the inferred optimal *K* using the program CLUMPP 1.1.2 (Jakobsson & Rosenberg, 2007) and DISTRUCT 1.1 (Rosenberg, 2004). To explore substructure in the dataset, STRUCTURE analysis was re‐run independently for each first‐order cluster identified with the first run across the study area (Pritchard & Wen, 2004).

### 2.5 Spatial genetic regression using Moran's eigenvector maps: MEMGENE

MEMGENE variables reflecting spatial genetic structure are the predicted values of a multivariate regression (redundancy analysis) between genetic distance and the spatial distance among individuals. These variables are extracted using “mgQuick” function (see Galpern et al. (2014) for details) in R with 1,000 forward permutations. The explanatory value of inferred spatial genetic patterns (represented by each MEMGENE axis) is estimated using regression analysis. Similar to STRUCTURE, MEMGENE analysis was conducted for the full study area followed by independent runs across inferred first‐order clusters.

### 2.6 Population genetics

Pairwise *F*_ST _was calculated among genetic clusters identified by STRUCTURE and MEMGENE using SPAGeDi version 1.5 (Hardy & Vekemans, 2002). MIGRATE software version 3.6 was used to measure effective number of migrants per generation to and from genetic clusters, with values averaged over 10 runs (Beerli, 2006; Beerli & Felsenstein, 2001). While boreal caribou are not migratory, assessing number of migrants per generation across genetically differentiated groups can help characterize gene flow in result of one‐way dispersal events for individuals. Expected heterozygosity (*H*_e_), observed heterozygosity (*H*_o_), number of alleles across loci (*A*), allelic richness (*A*_r_), and individual inbreeding coefficient (*F*_IS_) along with statistical significance were further calculated using SPAGeDi. Each cluster was further tested for allele frequency deviation from Hardy–Weinberg equilibrium (HWE) and linkage equilibrium (LE) using the probability test in GENEPOP version 4.2 (Rousset, 2008). Sequential Bonferroni corrections were applied across tests to remove error due to significance by chance (Rice, 1989).

### 2.7 Population genetics: isolation by distance

The relationship between geographical and genetic dissimilarity among individuals across the study area was evaluated using the Mantel test in software GenAlEx (Peakall & Smouse, 2006). IBD was also tested separately for male and female individuals to determine whether levels of spatial autocorrelation differed for each sex. The IBD model was additionally tested among individuals within each delineated second‐order genetic cluster. A Mantel correlogram using R package vegan (Oksanen et al., 2017) was used to identify the spatial extent to which IBD remains across the study area for males and females.

### 2.8 Landscape genetics: MEMGENE landscape analysis

MEM spatial variables were generated from models of hypothesized landscape resistance with distance between individuals measured using least‐cost paths. These resistance distances were then tested against spatial genetic patterns weighed by genetic dissimilarity within a regression framework. This analysis quantifies the proportion of genetic variation attributed to landscape‐specific spatial variation (i.e., IBR). Due to different levels of anthropogenic disturbance and general landscape heterogeneity across the study area, we tested landscape effects on genetic variation separately for each first‐order cluster identified by STRUCTURE and MEMGENE. Landscape variables hypothesized to cause landscape resistance to gene flow for boreal caribou across the study area included main roads, water bodies, and recent wildfires. It is known that various anthropogenic habitat disturbance features affect boreal caribou movements and distribution (e.g., Dyer, O'Neill, Wasel, & Boutin, 2001); however, we limited our analyses to evaluating the main roads. Each landscape variable was tested threefold as a univariate model under three cost values (10, 50, and 100). The cost value for the univariate model explaining the highest proportion of spatial variation for each variable was then used to create four optimized models that combined landscape variables. Vector shapefile and attribute data for water bodies were supplied by GeoGratis Natural Resources Canada and main roads (highways and provincial roads) by Statistics Canada National Road Network. A 500‐m buffer was included in the roads feature. Vector shapefile and attribute data for wildfires were supplied by the Canadian National Fire Database and were edited to only include a 50‐year period from 1964 to 2013. Straight line Euclidean distance in relation to genetic dissimilarity patterns, reflecting a model of IBD, was also tested by assigning a value of one to each cell in a raster surface of the study area. Isolation‐by‐barrier models were not tested as there are no large landscape features predicted to create barriers to gene flow for boreal caribou across the study area.

### 2.9 Management unit analysis: MEMGENE variation partitioning

To determine population boundaries that best maintained spatial patterns of genetic variation, variation partitioning by redundancy analysis was performed using “varPart” function in R package vegan. Explanatory variables in this multivariate regression (redundancy analysis) were a matrix of MEM spatial variables (mgQuick results) describing spatial components of genetic variation and a matrix of dummy explanatory variables coding the polygon membership of samples. The latter partitioned the sampling locations into hypothesized MUs across the study area. Four hypothesized population boundary scenarios were tested delineating the study area into five units. Only scenarios with the same number of units are directly comparable in this analysis, as there is no method available to penalize the addition of dummy variables representing unit membership that will inflate the proportion of variance explained. MU delineations were based on second‐order STRUCTURE (Scenario 1) and MEMGENE (Scenario 2) results, as well as local populations outlined in the federal recovery strategy while limiting partitioning of Manitoba into North and South ranges (Scenario 3; Figure 3). Finally, Scenario 4 combined local populations and STRUCTURE and MEMGENE results for the study area.

**Figure 4:**
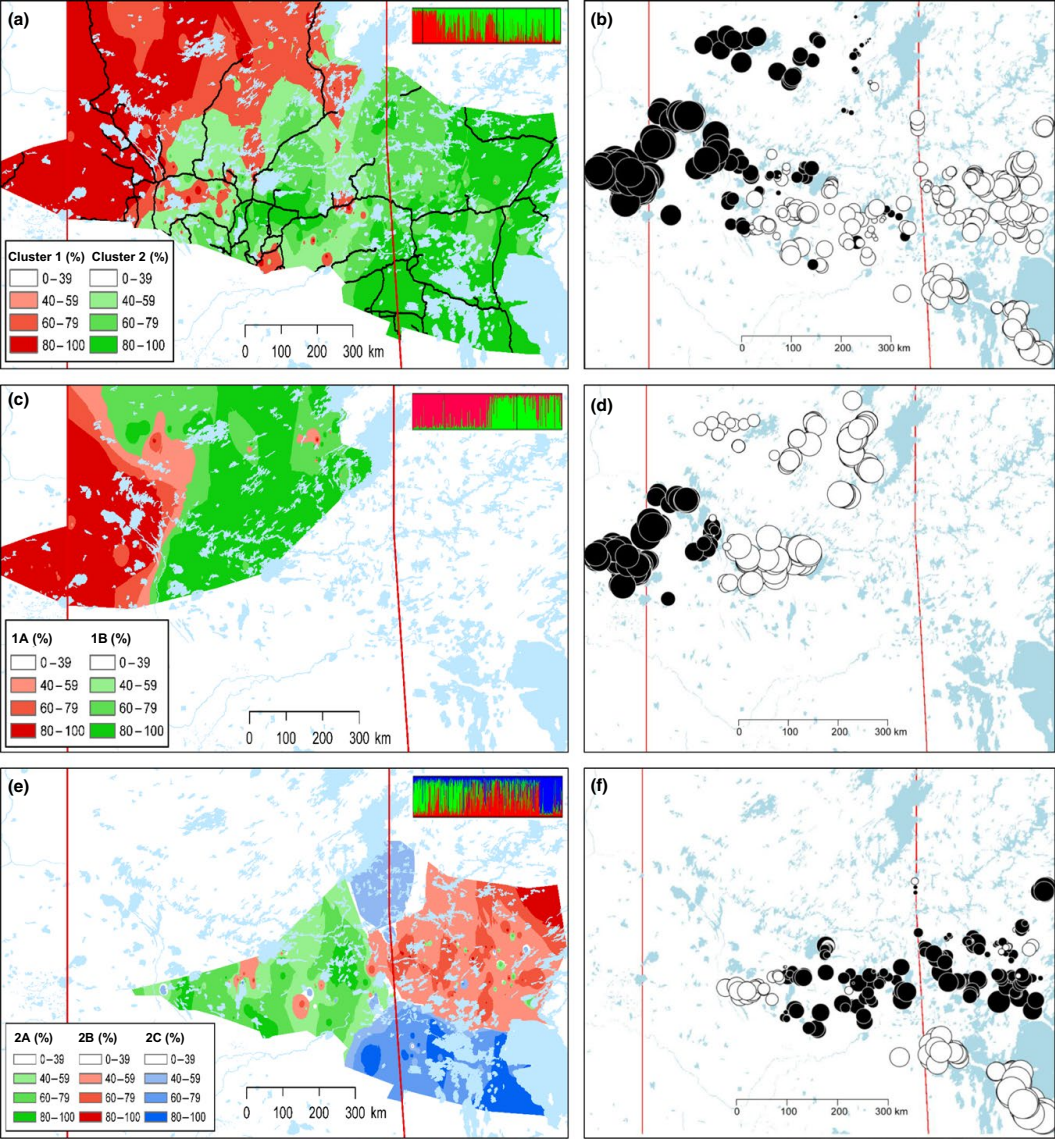
STRUCTURE (left) and MEMGENE (right) cluster assignment of individuals superimposed over the study area. Analyses included the (a & b) full study area and each first‐order cluster: (c & d) Cluster 1 and (e & f) Cluster 2, revealing different scales of spatial genetic variation. Bar plots on top right corners of STRUCTURE maps represent cluster assignments with colors corresponding to clusters spatially delineated using interpolated distance weights (legends included). Different colored and sized dots in MEMGENE maps similarly reflect cluster assignment. Black lines (a) indicate main roads

## 3 RESULTS

### 3.1 Individual identification

In eastern Alberta and Saskatchewan, 1,325 fecal samples and 88 blood blot samples were collected and genotyped at nine microsatellite loci. A total of 441 females, 213 males, and 43 individuals of unknown sex were identified as unique. An additional 524 unique genotypes (332 females, 146 males, and 46 unknown) from western Manitoba (Klütsch et al., 2016) were used in the analysis, bringing the total number of unique genotypes to 1,221 across the study area.

### 3.2 Population genetic structure: STRUCTURE

STRUCTURE analysis identified *K* = 2 at the first order of genetic structure separating the study area into West (Cluster 1) and East (Cluster 2) (Figure 4a). Further partitioning was present at *K* = 4 with breaks in gene flow found in western Saskatchewan, along the Saskatchewan and Manitoba provincial boundary, and with further partitioning in western Manitoba (Supporting Information Figures S1 and S2). High levels of admixture were found across the study area with admixture being highest in the center of the study area in Saskatchewan where genetic discontinuation was detected with first‐order clustering (Figure 4a). Further substructure was identified by analyzing each first‐order cluster and revealed two genetic clusters in Cluster 1 separating Cold Lake and western Saskatchewan from north‐central Saskatchewan (Figure 4c) and three clusters in Cluster 2 separating southeastern Saskatchewan, western Manitoba, and the Bog/Interlake area in south‐western Manitoba (Figure 4e). The partitioning of second‐order clusters supported *K* = 2 and *K* = 4 clustering results for the full study area.

### 3.3 Spatial genetic regression using Moran's eigenvector maps: MEMGENE

MEMGENE analysis revealed that the total amount of genetic variation explained by spatial patterns across the study area was 14% and was mainly explained by the first two axes. The first axis of variation found genetic divergence in central Saskatchewan, separating the east and west of the study area (Figure 4b) similar to STRUCTURE results at *K* = 2 (Figure 4a). The second axis of variation found genetic divergence in western Saskatchewan connecting the southwest of the province with Cold Lake and further genetic divergence between south‐western Manitoba (Bog/Interlake) and the remaining study area (Supporting Information Figure S2). For Cluster 1 only, 9% of genetic variation was explained by the first axis separating Cold Lake and western Saskatchewan from north‐central Saskatchewan (Figure 4d) similar to second‐order STRUCTURE results (Figure 4c). For Cluster 2 only, 12% of genetic variation was explained by the first axis separating the Bog/Interlake area and combining eastern Saskatchewan and western Manitoba, leaving a small cluster in the south‐central region of Saskatchewan where Prince Albert National Park is found (Figure 4f).

### 3.4 Population genetics

Pairwise *F*_ST _values indicated relatively low genetic differentiation across higher level cluster pairs (*F*_ST_ <0.05; Table 1). All second‐order cluster pairwise *F*_ST_ comparisons were significant (*p* < 0.001) and ranged from 0.01 to 0.08 (Table 1). Cluster 2C (Bog/Interlake area) was the most genetically differentiated cluster with the highest *F*_ST_ (Table 1). MIGRATE analysis indicated varying levels of dispersal across second‐order cluster pairs with migrants per generation ranging from 0.20 to 16.45 migrants per generation across all genetic clusters (Table 2). Dispersal was evident across the full extent of the study area and was highest from and between Cluster 2A in southeastern Saskatchewan and Cluster 2B in northwestern Manitoba (Table 2). Dispersal from and toward Cluster 1A (Cold Lake) and Cluster 2C (Manitoba South) in the peripheries of the study area was lowest (Table 2). Loci deviating from HWE included one locus for Cluster 1 (RT6) and five loci for Cluster 2 (Supporting Information Table S1). Within second‐order clusters, Clusters 1A, 1B, and 2C had all nine loci in HWE, while Clusters 2A and 2B had three and one loci in disequilibrium, respectively (Supporting Information Table S1). Additional results for genetic diversity indices can be found in Supporting Information Table S1.

**Table 1:** Pairwise *F*_ST_ values for the two first‐order genetic clusters indicated by number (above) and five second‐order genetic clusters indicated by number and letter (below) across the study area. Cluster delineation is depicted in Figure 4

Cluster		1	2		
1		—	0.02		
2		—	—		
	**1A**	**1B**	**2A**	**2B**	**2C**
1A	—	0.03	0.04	0.06	0.08
1B	—	—	0.01	0.02	0.05
2A	—	—	—	0.01	0.04
2B	—	—	—	—	0.04
2C	—	—	—	—	—

Note

All values are significant (*p* < 0.001).

**Table 2:** MIGRATE results indicating direct dispersal (number of migrants per generation) from (column) and to (row) each first‐order genetic cluster indicated by number and each second‐order genetic cluster indicated by number and letter. Cluster delineation is depicted in Figure 4

Migration from/to	1A	1B	2A	2B	2C
1A	—	0.20	0.38	0.40	0.16
1B	6.54	—	12.49	16.45	2.86
2A	9.47	8.35	—	12.42	3.82
2B	7.80	9.59	11.42	—	6.36
2C	0.22	0.47	0.32	0.56	—

### 3.5 Population genetics: isolation by distance

Isolation by distance was significant across the full study area and all first‐ and second‐order clusters (*p* < 0.005; Table 3), providing additional evidence that spatial proximity among individuals explains some of the genetic variation within clusters. IBD did not differ for males and females across the study area (Table 3), and spatial autocorrelation remained significant up to distances just below 1,500 km for both sexes (Supporting Information Figure S3). The correlation between geographic and genetic distance was highest for Cluster 2C (Mantel *r* = 0.13; Table 3), while the correlation between geographic and genetic distance was lowest for Cluster 1B (Mantel *r = *0.07; Table 3).

**Table 3:** Isolation by distance (IBD) results with Mantel *r* and *p* values showing the linear relationship between geographic distance and genetic distance among individuals across the full study area, among female and male individuals separately across the full study area, and among all individuals across each genetic cluster. Sample size (*n*) of each area is shown. Cluster delineation is depicted in Figure 4

Area	Mantel *r*	*p* value	*n*
Full study area	0.11	0.001	1,221
Full female	0.11	0.001	773
Full male	0.12	0.001	359
Cluster 1	0.12	0.001	393
Cluster 2	0.08	0.001	828
Cluster 1A	0.08	0.004	196
Cluster 1B	0.07	0.002	197
Cluster 2A	0.08	0.001	296
Cluster 2B	0.12	0.001	405
Cluster 2C	0.13	0.001	127

### 3.6 Landscape genetics: MEMGENE landscape analysis

All models were significant in explaining landscape resistance across the study area (Table 4). Following model optimization (Supporting Information Table S2), the models including roads remained the best models. In the more western Custer 1, the best model required a higher cost value for roads to be significant (cost of 100) compared with the more eastern Cluster 2 (cost of 10), indicating a stronger effect of roads in the eastern than the western part of the study area. Water was as important as roads in explaining genetic variation in Cluster 2, while fire was the least important variable in both clusters and did not explain more variation than IBD.

**Table 4:** MEMGENE landscape analysis results for each first‐order cluster (Cluster 1 and Cluster 2; delineation depicted in Figure 5). Seven optimized landscape models in addition to Euclidean distance (IBD) were tested for each cluster. The table describes the proportion of variation in genetic distance that can be explained by [abc] spatial predictors (selected MEM eigenvectors), [a] spatial patterns in the landscape model, [c] coordinates, [b] confounded patterns in the landscape model and coordinates, and finally [d] residual (nonspatial) patterns. *p *[abc], *p *[a], and *p *[c] represent the significance (*p* value) of each calculated proportion

	[abc]	*p *[abc]	[a]	*p *[a]	[c]	*p *[c]	[b]	[d]
Cluster 1								
IBD	0.10	0.001	0.05	0.001	0.01	0.001	0.04	0.90
Roads (100)	0.12	0.001	0.08	0.001	0.01	0.001	0.04	0.88
Water (10)	0.11	0.001	0.07	0.001	0.01	0.001	0.04	0.89
Fire (10)	0.10	0.001	0.05	0.001	0.01	0.001	0.04	0.91
Roads and water	0.12	0.001	0.07	0.001	0.003	0.014	0.05	0.88
Roads and fire	0.11	0.001	0.07	0.001	0.01	0.001	0.04	0.89
Water and fire	0.10	0.001	0.05	0.001	0.01	0.001	0.04	0.90
Roads, water and fire	0.11	0.001	0.06	0.001	0.01	0.001	0.04	0.89
Cluster 2								
IBD	0.12	0.001	0.09	0.001	0.003	0.001	0.03	0.88
Roads (10)	0.13	0.001	0.10	0.001	0.002	0.001	0.03	0.87
Water (10)	0.13	0.001	0.10	0.001	0.003	0.001	0.03	0.87
Fire (10)	0.12	0.001	0.09	0.001	0.003	0.001	0.03	0.88
Roads and water	0.13	0.005	0.10	0.005	0.003	0.005	0.03	0.87
Roads and fire	0.13	0.005	0.09	0.005	0.002	0.005	0.03	0.87
Water and fire	0.12	0.005	0.08	0.005	0.01	0.005	0.03	0.88
Roads, water, and fire	0.11	0.005	0.08	0.005	0.01	0.005	0.03	0.89

**Figure 5:**
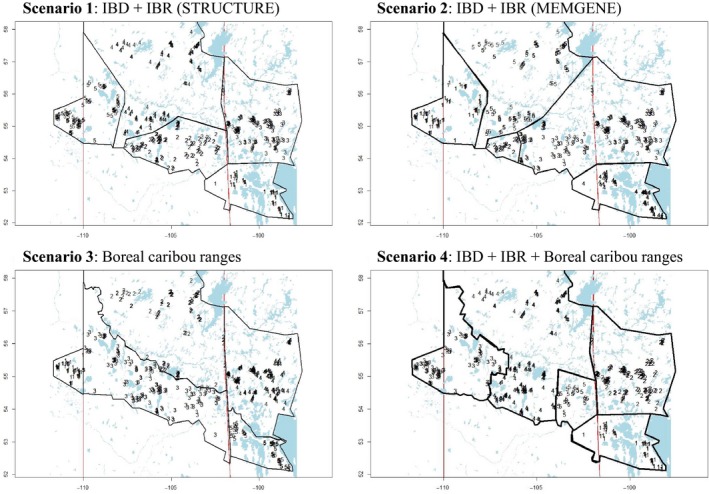
Four hypothesized management unit (MU) scenarios tested to determine the population delineation that best reflects genetic variation of boreal caribou across the study area. MU delineation was based on varying levels of IBD and IBR reflected in second‐order STRUCTURE results, (Scenario 1) and second‐order MEMGENE results, (Scenario 2) coarse‐scale local populations outlined in the federal recovery strategy (Scenario 3; Environment Canada, 2012), and a combination of boreal caribou ranges and genetic clustering results (Scenario 4). Samples are numbered in each map according to MU assignment (n = 5)

### 3.7 Management unit analysis: MEMGENE variation partitioning

The MU scenarios explaining the highest proportion of spatial genetic patterns across the study area included both Scenario 1 (reflecting second‐order STRUCTURE results) and Scenario 2 (reflecting second‐order MEMGENE results), followed closely by Scenario 4 (combining local populations and observed genetic structure) (Table 5 [a]; Figure 5), Scenario 3 (reflecting local populations) best reflected the spatial proximity of individuals only (Table 5 [b]; Figure 5). The MU scenario explaining the highest proportion of spatial genetic patterns in result of both MU boundaries and spatial proximity of individuals was Scenario 1, followed by Scenario 2, Scenario 4, and lastly Scenario 3 (Table 5 [c]; Figure 5).

**Table 5:** Adjusted *R* ^2^ values indicating the proportion of the spatial genetic pattern in the first MEMGENE axis across the study area that can be explained by the [a] MU scenario, [b] spatial proximity of individuals, [c] MU scenario and spatial proximity of individuals combined, and [d] residual patterns. Each scenario is depicted in Figure 5

Scenario	[a]	[b]	[c]	[d]
1	0.21	0.03	0.49	0.28
2	0.21	0.05	0.46	0.28
3	0.16	0.14	0.37	0.33
4	0.20	0.08	0.44	0.29

Note

All fractions are significant (*p* < 0.001).

## 4 DISCUSSION

### 4.1 IBD and population structure

The significance of IBD across the study area and within each genetic cluster supported the hypothesis that boreal caribou maintain a natural clinal pattern of genetic structure. Spatial autocorrelation persisted across a large spatial scale supporting previous telemetry‐based studies that reported relatively large home range sizes for the ecotype (McLoughlin et al., 2004; Rettie & Messier, 2001). The similarity in strength of IBD across large scales for both males and females may be explained by long‐distance dispersing males looking for mates, the search for home ranges, and calving sites by females as suitable habitat is depleted or burned, as well as general avoidance of anthropogenic disturbance by both sexes (Dalerum, Boutin, & Dunford, 2007; Faille et al., 2010). IBD across the study area was also reflected in measures of *F*_ST_ and number of migrants per generation, which revealed higher genetic differentiation and lower levels of dispersal between clusters found further apart compared with neighboring central clusters. Additionally, the two central clusters Cluster 2A and Cluster 2B had low levels of genetic differentiation and were the only second‐order clusters found with loci deviating from HWE, providing further evidence of immigration (i.e., dispersal) and non‐random mating, and suggesting that individuals in these clusters do not form demographically independent groups. The high level of dispersal and low genetic differentiation identified between Cluster 2A in southeastern Saskatchewan and Cluster 2B in the Manitoba North range supported findings by Ball et al. (2010) that identified high genetic connectivity (*F*_ST _=0.03) between these areas. Additionally supported by our results was the genetic connectivity previously found in the Interlake and Bog regions in Manitoba and extending into southeastern Saskatchewan (Ball et al., 2010). Following partitioning of the study area, we found IBD was stronger in the western part of the study area that is less fragmented by anthropogenic disturbance (Mantel *r* = 0.12), but IBD was still moderate in the eastern part of the study area and within clusters with relatively high levels of landscape fragmentation (i.e., Cluster 2A; Mantel *r* = 0.08). The strong level of IBD present within and among clusters suggests that dispersal is based heavily on geographic distance rather than social behavior that would support demographically and structurally independent groups. Because the boreal caribou ecotype is genetically different from neighboring barren‐ground and eastern migratory ecotypes (see Klütsch et al., 2016 for more information), the effect of migratory pathways is not considered to be an important factor in the characterization of genetic structure in boreal caribou. Different evolutionary lineages may additionally play a role in shaping genetic structure; however, Klütsch, Manseau, and Wilson (2012) identified that the majority of boreal caribou found west of Manitoba consist of the A2 haplotype; therefore, genetic structure is likely not impacted by different lineages of glacial refugia.

MEMGENE, a visualization approach based on the predicted values of a spatial regression rather than on cluster assignment, was able to confirm areas of genetic discontinuity in our study while further identifying finer‐scale genetic variation not detected by STRUCTURE. MEMGENE avoids the requirement that spatial genetic clusters are in HWE, which is central to the algorithm in the program STRUCTURE (Pritchard et al., 2000) but may not be common in wild populations with high levels of admixture caused by dispersal (Waples & Gaggiotti, 2006). Previous studies on other vagile species have similarly found that MEMGENE identified comparable patterns while revealing finer‐scale genetic variation not detected with other spatially explicit methods, allowing for population‐level structure to be carefully assessed and cryptic spatial patterns to be revealed (Galpern et al., 2014; Richardson et al., 2017; Robertson, Fletcher, & Austin, 2017). The regression framework MEMGENE uses further permitted different orthogonal axes representing overlapping spatial genetic patterns to be directly compared to aid in the detection of hierarchical structure characterized by multiple layers of discontinuity. For example, genetic partitioning was detected despite low genetic differentiation between the two coarse‐scale clusters found across the study area (FST = 0.02); however, levels of admixture and fine‐scale differentiation identified with STRUCTURE and MEMGENE indicated that partitioning may be confounded by overlaying IBD/IBR processes. Assessing finer‐scale structure using both STRUCTURE and MEMGENE allowed for localized boundaries that reflect patterns of genetic variation of boreal caribou across the study area to be identified and for the effects of natural and anthropogenic features that influence spatial patterns to be incorporated as margins of connectivity into population‐level delineation.

### 4.2 Landscape effects on gene flow

Further evaluating IBD within a landscape genetics framework aided in explaining many of the spatial genetic discontinuities detected by STRUCTURE and MEMGENE while disentangling the confounding effects of IBR caused by landscape heterogeneity. For example, roads can help explain some of the genetic discontinuity detected between clusters, such as the highway running north between Clusters 1A and 1B in Saskatchewan and the high density of high‐use roads and highways in south‐central Saskatchewan surrounding Prince Albert National Park. Furthermore, the strong genetic differentiation in the Manitoba South range, known in this study as Cluster 2C, supports previous studies that found roads and waterbodies in this region to restrict boreal caribou dispersal (Ball et al., 2010; Fall, Fortin, Manseau, & O'Brien, 2007). Wildfire was not as important as roads and waterbodies at explaining spatial genetic variation of boreal caribou, which can be explained by strong site fidelity to areas following a wildfire even with over 70% of direct disturbance to a home range (Dalerum et al., 2007).

The contributions of both IBD and natural and anthropogenic landscape features in restricting gene flow were significant, suggesting that the relative importance of these processes in driving boreal caribou population structure can be difficult to partition. Disentangling the effects of IBD and IBR is difficult in regions presenting limiting resistance to gene flow (e.g., Ruiz‐Gonzalez, Cushman, Madeira, Randi, & Gómez‐Moliner, 2015; Kierepka & Latch, 2016) and when the competing models are strongly correlated (Cushman & Landguth, 2010; Shirk, Landguth, & Cushman, 2017; Zeller et al., 2016). We attempted to reduce confounding effects and strong correlation among competing models by testing models with only three landscape variables that were strong candidates for reducing gene flow. Success in separating drivers of genetic variation is also commonly scale‐dependent (Cushman & Landguth, 2010; Galpern, Manseau, & Wilson, 2012; Landguth & Schwartz, 2014), and while analyzing each coarse‐scale cluster separately helped reveal regional landscape effects on gene flow, a finer‐scale analysis could additionally help detect factors that may be more important in more localized regions of the study area. Further, despite the expected correlation found between areas with genetic discontinuity and the presence of roads and waterbodies with our IBR models, a higher IBR signal may not yet be detectable across the study area due to time lags in genetic differentiation. Fragmentation and landscape resistance may take many generations to become evident across genetic data as the effects may be decelerated by even low levels of gene flow (Landguth et al., 2010; Epps & Keyghobadi, 2015).

### 4.3 Management unit delineation

Analyzing multiple MU scenarios provided a comprehensive analysis of population‐level delineation based on patterns of genetic variation and revealed that current boreal caribou population units identified in the federal recovery strategy across our study area do not capture population genetic structure of the ecotype. Our analyses of genetic connectivity across the study area further confirmed that discrete population boundaries do not exist. Most evidently, the localized populations in the Manitoba North range have been found to be too genetically connected to be considered as independent herds. Also, range division of north and south Saskatchewan, based on ecozone boundaries, does not reflect the genetic connectivity results captured in our analyses. We therefore recommend that, together with other ecological factors affecting boreal caribou conservation and recovery (e.g., Environment Canada, 2011, 2012 ), the delineation of boreal caribou conservation units be informed by population genetic structure to best maintain, or restore, long‐term genetic connectivity across the landscape. Similarly, landscape effects on gene flow should be incorporated into conservation and management planning, and special care should be taken to mitigate the effects of roads in reducing landscape connectivity for the ecotype.

## 5 CONCLUSIONS

We showed that IBD played a dominant role in shaping spatial patterns of genetic variation across the landscape. Landscape genetics was useful in explaining the genetic discontinuity detected and the contribution of natural and anthropogenic landscape variables in restricting gene flow. As a result, estimating the relative contribution of IBD and IBR on genetic differentiation helped characterize connectivity at and below the population level, revealing a baseline clinal pattern of genetic variation across the study area.

Strong IBD results provided challenges for population‐level delineation under assumptions of spatial and demographic independence as defined for a local population in the federal recovery strategy. The combined resistance effect of the landscape variables evaluated here, and IBD, was not large enough to cause strong breaks in gene flow and higher level divergence that are needed to classify discrete populations or MUs (Palsbøll et al., 2007; Waples & Gaggiotti, 2006). MEMGENE was more sensitive at detecting weak patterns of genetic variation that were not detected by STRUCTURE but revealed localized IBD and IBR effects on gene flow. Additionally, using Moran's eigenvectors to evaluate landscape effects on gene flow and compare MU scenarios was a valuable tool for validating the delineation of cluster boundaries that are sensitive to clinal processes. Despite the challenges and pressures surrounding the delineation of discrete localized population boundaries for management planning, these approaches can be used in future applications to assist in MU delineation that maintains natural patterns of gene flow for highly vagile wildlife species across anthropogenically disturbed landscapes and to prevent further loss in genetic connectivity that may not be easily restored.

## CONFLICT OF INTERESTS

None declared.

## AUTHOR CONTRIBUTIONS

MM and PJW conceived and designed the project. Data collection was organized by DH, MM, TT, and PDM. PP analyzed the data. Extension of MEMGENE methods and support for data analysis were provided by PG. Reagents, materials, and other tools for analysis were contributed by PJW. All authors contributed critically to drafts and gave final approval for publication.

## DATA ACCESSIBILITY

Data available from the Dryad Digital Repository: https://doi.org/10.5061/dryad.7k2g187
