# Supplementary material for: Partitioning drivers of spatial genetic variation for a continuously distributed population of boreal caribou: Implications for management unit delineation
Source: Ecol Evol. 2018 Dec 14;9(1):141–53. doi: 10.1002/ece3.4682 (PMC6342118; doi:10.1002/ece3.4682)
Supplement: Supplementary file 1 [file ECE3-9-141-s001.docx]

**Supporting Information**

**Partitioning drivers of spatial genetic variation for a continuously-distributed population of boreal caribou: implications for management unit delineation**

**Priadka et al.**

This file contains supplementary tables and figures

**Table S1**. Descriptive statistics including loci deviating from Hardy-Weinberg equilibrium (HWE), expected heterozygosity (*H*_e_), observed heterozygosity (*H*_o_), number of alleles across loci (*A*), allelic richness (*A*_r_), and inbreeding coefficient (*F*_IS_) for the two first-order genetic clusters (indicated by number) and five second-order genetic clusters (indicated by number and letter) delineated across the study area (delineation shown in Figure 4 in manuscript).

| Cluster | HWE (across 9 loci) | *H*_e_ | *H*_o_ | *A* | *A*_r_ | *F*_IS_ |
| --- | --- | --- | --- | --- | --- | --- |
| 1 | RT6* | 0.778 | 0.739 | 16.00 | 15.78 | 0.051** |
| 2 | BM848**, RT24**, RT30**, RT7**, RT9* | 0.747 | 0.688 | 15.11 | 13.83 | 0.078** |
| 1A | - | 0.760 | 0.738 | 13.11 | 12.10 | 0.028 |
| 1B | - | 0.775 | 0.740 | 13.44 | 12.48 | 0.045** |
| 2A | RT24**, RT30**, RT7** | 0.743 | 0.699 | 13.22 | 11.71 | 0.059** |
| 2B | RT30** | 0.744 | 0.689 | 12.22 | 10.62 | 0.075** |
| 2C | - | 0.692 | 0.662 | 9.00 | 8.91 | 0.043 |
| **P* < 0.005 ***P* < 0.001 | | |  |  |  |  |

**Table S2**. All landscape models tested using MEMGENE landscape analysis for variables roads, water and fire under different hypothesized cost values (10, 50 and 100) for Cluster 1 and Cluster 2 (delineation shown in Figure 4 in manuscript). The model with the cost value that performed best for each variable (indicated in bold) was used to create four optimized landscape models testing combinations of all variables (see Table 4 in manuscript). Each value in the table describes the proportion of variation in genetic distance that can be explained by [abc] spatial predictors (selected MEM eigenvectors), [a] spatial patterns in the landscape model, [c] coordinates, [b] confounded patterns in the landscape model and coordinates, and finally [d] residual (non-spatial) patterns. *P*[abc], *P*[a] and *P*[c] represent the significance (*P* value) of each calculated proportion.

| Cluster 1 | [abc] | *P*[abc] | [a] | *P*[a] | [c] | *P*[c] | [b] | [d] |
| --- | --- | --- | --- | --- | --- | --- | --- | --- |
| roads (10) | 0.106 | 0.001 | 0.057 | 0.001 | 0.005 | 0.001 | 0.044 | 0.894 |
| roads (50) | 0.121 | 0.001 | 0.072 | 0.001 | 0.005 | 0.001 | 0.044 | 0.879 |
| **roads (100)** | **0.124** | **0.001** | **0.075** | **0.001** | **0.005** | **0.001** | **0.044** | **0.876** |
| **water (10)** | **0.114** | **0.001** | **0.065** | **0.001** | **0.005** | **0.001** | **0.044** | **0.886** |
| water (50) | 0.106 | 0.001 | 0.057 | 0.001 | 0.006 | 0.001 | 0.043 | 0.894 |
| water (100) | 0.106 | 0.001 | 0.057 | 0.001 | 0.006 | 0.001 | 0.043 | 0.894 |
| **fire (10)** | **0.095** | **0.001** | **0.046** | **0.001** | **0.008** | **0.001** | **0.041** | **0.905** |
| fire (50) | 0.088 | 0.001 | 0.039 | 0.001 | 0.014 | 0.001 | 0.035 | 0.912 |
| fire (100) | 0.084 | 0.001 | 0.035 | 0.001 | 0.018 | 0.001 | 0.031 | 0.916 |
| Cluster 2 | [abc] | P[abc] | [a] | P[a] | [c] | P[c] | [b] | [d] |
| **roads (10)** | **0.132** | **0.001** | **0.099** | **0.001** | **0.002** | **0.001** | **0.031** | **0.868** |
| roads (50) | 0.130 | 0.001 | 0.097 | 0.001 | 0.002 | 0.001 | 0.031 | 0.870 |
| roads (100) | 0.118 | 0.001 | 0.085 | 0.001 | 0.002 | 0.002 | 0.031 | 0.882 |
| **water (10)** | **0.131** | **0.001** | **0.098** | **0.001** | **0.003** | **0.001** | **0.030** | **0.869** |
| water (50) | 0.129 | 0.001 | 0.095 | 0.001 | 0.004 | 0.001 | 0.030 | 0.871 |
| water (100) | 0.129 | 0.001 | 0.096 | 0.001 | 0.004 | 0.001 | 0.029 | 0.871 |
| **fire (10)** | **0.124** | **0.001** | **0.091** | **0.001** | **0.003** | **0.001** | **0.031** | **0.876** |
| fire (50) | 0.099 | 0.001 | 0.066 | 0.001 | 0.008 | 0.001 | 0.025 | 0.901 |
| fire (100) | 0.077 | 0.001 | 0.044 | 0.001 | 0.012 | 0.001 | 0.021 | 0.923 |
|  |  |  |  |  |  |  |  |  |

**Figure S1**. Delta K graph for STRUCTURE results across the full study area.


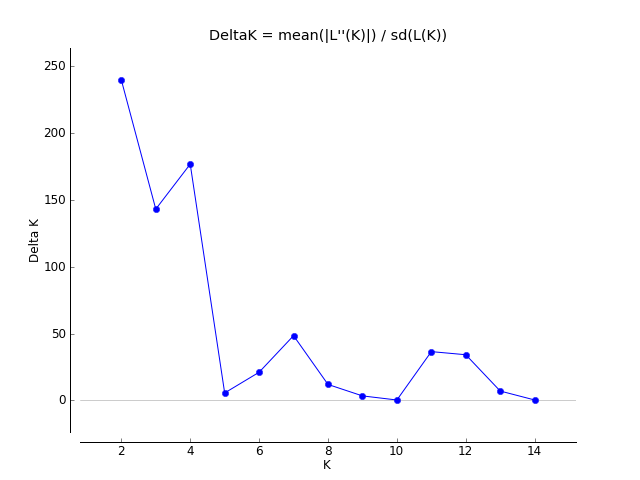


**Figure S2**. STRUCTURE *K* = 4 (left) and second axis MEMGENE (right) results for the full study area. Bar plots on top right corner of the STRUCTURE map represents cluster assignments for individuals with colours corresponding to spatially delineated clusters delineated spatially using Interpolated Distance Weights (IDW). Colour gradients for each cluster reflect strength of cluster assignment (legend included). Black lines indicate main roads.

**Figure S3**. Mantel correlograms for female (above) and male (below) boreal caribou across the full study area.
